# Novel insights into the rehabilitation of memory post acquired brain injury: a systematic review

**DOI:** 10.3389/fnhum.2014.00993

**Published:** 2014-12-16

**Authors:** Lauriane A. Spreij, Johanna M. A. Visser-Meily, Caroline M. van Heugten, Tanja C. W. Nijboer

**Affiliations:** ^1^Department of Experimental Psychology, Helmholtz Institute, Utrecht UniversityUtrecht, Netherlands; ^2^Brain Center Rudolf Magnus, Center of Excellence for Rehabilitation Medicine, University Medical Center Utrecht, and De Hoogstraat RehabilitationUtrecht, Netherlands; ^3^Department of Psychiatry and Neuropsychology, Faculty of Health, Medicine and Life Sciences, Maastricht UniversityMaastricht, Netherlands

**Keywords:** acquired brain injury, memory, remediation-oriented technique, virtual reality, computer-based cognitive retraining, non-invasive brain stimulation

## Abstract

**Objective:** Acquired Brain Injury (ABI) frequently results in memory impairment causing significant disabilities in daily life and is therefore a critical target for cognitive rehabilitation. Current understanding of brain plasticity has led to novel insights in *remediation-oriented* approaches for the rehabilitation of memory deficits. We will describe 3 of these approaches that have emerged in the last decade: Virtual Reality (VR) training, Computer-Based Cognitive Retraining (CBCR) and Non-Invasive Brain Stimulation (NBS) and evaluate its effectiveness.

**Methods:** A systematic literature search was completed in regard to studies evaluating interventions aiming to improve the memory function after ABI. Information concerning study content and reported effectiveness were extracted. Quality of the studies and methods were evaluated.

**Results:** A total of 786 studies were identified, 15 studies met the inclusion criteria. Three of those studies represent the VR technique, 7 studies represent CBCR and 5 studies NBS. All 3 studies found a significant improvement of the memory function after VR-based training, however these studies are considered preliminary. All 7 studies have shown that CBCR can be effective in improving memory function in patients suffering from ABI. Four studies of the 5 did not find significant improvement of the memory function after the use of NBS in ABI patients.

**Conclusion:** On the basis of this review, CBCR is considered the most promising novel approach of the last decade because of the positive results in improving memory function post ABI. The number of studies representing VR were limited and the methodological quality low, therefore the results should be considered preliminary. The studies representing NBS did not detect evidence for the use of NBS in improving memory function.

## Introduction

Memory impairment is a common consequence of Acquired Brain Injury (ABI) which causes significant disabilities and is therefore a critical target for cognitive rehabilitation (Hall et al., 2005; Yip and Man, 2013). ABI is defined as damage to the brain that occurs after birth and is not related to congenital disorders, developmental disabilities, or processes that progressively injure the brain. The majority of ABI is caused by Traumatic Brain Injury (TBI) and hemorrhagic or ischemic stroke (Holmqvist et al., 2009). Within the *TBI* population, the percentage of people suffering from some form of memory impairment ranges from 20 to 79%, depending on the severity of the (closed) head trauma, the time of measurement, and the instruments used. Even after 1 year, 4 to 25% of TBI patients show some form of memory impairment (Cappa et al., 2011). The prevalence of memory dysfunction *post-stroke* varies from 23 to 55% in the first 3 months, which declines 1 year post-stroke to a percentage between 11 and 31% (Das Nair and Lincoln, 2007; Snaphaan and de Leeuw, 2007; Aben et al., 2013). Memory impairments can hamper independence in activities of daily living, as well as return to work, social participation and the overall quality of life (Fish et al., 2008). For this reason and the high prevalence of memory impairment after ABI, there is a urgent need for effective cognitive rehabilitation.

There are 2 main approaches within memory rehabilitation. First, *remediation* by restoration or retraining of the function; and second, *compensation* referring to any compensatory strategies, environmental modifications, and intact cognitive functions to overcome limitations in daily life. *Remediation* of the function is primarily due to some degree of spontaneous recovery (Cramer, 2008). The understanding of spontaneous recovery has been accompanied by the development of a wide range of therapeutic approaches that target brain repair by restoration. These can be referred to as *remediation*-oriented therapies, the aim of which is not to salvage threatened tissue but to promote restoration of function. Retraining of the function is based on the assumption that impaired memory will respond to mental exercise in the same manner as muscles respond to physical exercise and that repetitive training in 1 memory task may generalize to improved performance on other tasks within the same memory system (Brooks and Rose, 2003). The hypothesis is that the capacity of the function improves if the training is successful and does not depend on context or learning abilities (Björkdahl et al., 2013). Unfortunately, until recently there was little empirical evidence to indicate that these techniques are of much benefit to patients as any improvement on specific tasks practiced have not been found to generalize to other similar tasks (Brooks and Rose, 2003; Rees et al., 2007).

On the contrary, Rees et al. (2007) found strong evidence for the use of *compensation* for lost or deficient memory function. Therefore, most memory rehabilitation interventions focus on alleviating memory problems on functional level (i.e., level of activity in daily life), without necessarily improving the underlying memory function (Rees et al., 2007). Current memory treatment programs have focused on teaching patients the use of internal strategies (e.g., repeating, counting, face-name associations, categorizing, mental visualization or rhyming mnemonics) and/or external strategies (e.g., diaries, notebooks, to-do lists, electronic organizers, pagers) to help remembering and recalling information (Fish et al., 2008). In an updated review of evidence-based rehabilitation, Cicerone et al. (2011) recommended training in the use of external compensation strategies (including assistive technology) with direct application to daily activities as a practice guideline for individuals with moderate to severe memory impairment after TBI or stroke.

In brief, there has been little research showing that memory can be improved through *remediation*-oriented therapies and hence *compensation* approaches are the treatment choice. However, with the recently maturing fields in cognitive neurosciences, neuroplasticity shows greater promises then previously assumed and has yielded new interdisciplinary approaches (Miniussi et al., 2008). Neuroplasticity is the ability of the brain to create, strengthen, and modify neurological connections. It occurs at many levels from molecules to cortical reorganization. *Remediation*-oriented rehabilitation, based on neuroplasticity, can not only modify neural connections, but can also lead to functional relearning (Kimberley et al., 2010). This allows brain injured patients to relearn new knowledge and establish new skills (Li et al., 2013).

The current understanding of brain plasticity has led to novel insights in the rehabilitation of memory deficits. However, an overview of these insights is missing. The aim of this systematic review is to describe novel memory rehabilitation interventions based on *remediation*-oriented techniques post ABI and evaluate its effectiveness. This review will not include studies evaluating pharmacological intervention as pharmacological therapies were considered not suitable for targeting only the memory function without affecting other cognitive functions. We will describe 3 non-pharmacological approaches aiming at restoring the memory function that have emerged in the last decade: Virtual Reality (VR) training, Computer-Based Cognitive Retraining (CBCR) and Non-Invasive Brain Stimulation (NBS).

### Virtual Reality

Virtual environments represent many real-life situations and are programmed to record accurate measurements of the individual's performance assessing the underlying function (Brooks and Rose, 2003). VR is an interactive computer technology which creates the illusion of being in an artificial world. An fMRI study indicated that virtual-based environments are able to activate the related brain parts as in the real environment (You et al., 2005). The transfer of learned skills from VR training to real-life situations has been reported, which shows a high ecological validity (Brooks and Rose, 2003). VR is often used to obtain a realistic and controlled assessment of memory impairment in a rehabilitation setting (Brooks et al., 2004) However, the use of VR in rehabilitation is not only useful as an assessment tool, but also as the potential to offer a training method restoring the memory function.

### Computer-Based Cognitive Retraining

CBCR, based on intensive repetition, aims at improving cognitive skills needed to successfully receive sensory input, process information, and react without any use of external aids (Li et al., 2013). CBCR is available to the patient at home and offers stimulating tailored programs that can be modified to the individual's progress. Ample evidence is found suggesting CBCR is effective in the recovery of working memory (WM) (Olesen et al., 2004). Studies investigating CBCR in healthy participants showed that training can increase WM capacity and that training-induced changes in brain activity occur (Olesen et al., 2004; Westerberg et al., 2007). Additionally, training effects can be generalized to non-trained WM tasks, and to tests on attention, reasoning, and problem solving. Transfer of the training effects to non-trained WM tasks is consistent with the notion of training-induced plasticity in a common neural network for WM. The observed training effects suggest that WM training could be used as a *remediation* intervention for individuals for whom low WM capacity is a limiting factor in everyday life (Klingberg, 2010).

### Non-Invasive Brain Stimulation

Different neurophysiologic strategies to increase the activity of the injured brain area have been proposed mainly using Transcranial Magnetic Stimulation (TMS) and Transcranial Direct Current Stimulation (tDCS). TMS is based on the principle of electromagnetic induction and causes depolarization and hyperpolarization in the neurons. Lower frequencies of repetitive TMS is called repetitive Transcranial Magnetic Stimulation (rTMS), this is a train of TMS pulses delivered at constant intervals on the same intensity (low-frequency 1–4 Hz, high-frequency 5–10 Hz). rTMS presents the opportunity to interact even more effectively with cortical activity (Miniussi et al., 2008). Transcranial Direct Current Stimulation (tDCS) consists of placing 2 rubber electrodes on the scalp in order to allow a weak direct current to flow from anode to cathode. The electrical stimulus that reaches the brain is of enough intensity to modify the level of spontaneous neuronal excitability and activity by changing the resting membrane potential. tDCS is easier to apply and less expensive then TMS (Johansson, 2011).

Several studies emphasize the fact that interacting with cortical activity by cortical stimulation can positively affect cognitive performance and improve the rehabilitation potential (Miniussi et al., 2008). The therapeutic strategy of NBS consists of modulating an adaptive organization, allowing for the formation of functionally appropriate neural connections and enhancing behavioral recovery (Villamar et al., 2012). Preliminary evidence suggests that NBS may play a role in treating unilateral neglect (Nyffeler et al., 2009; Lim et al., 2010) and aphasia (Naeser et al., 2005; Szaflarski et al., 2011).

To summarize, the aim of this review is to provide an overview of the studies characterizing the most discussed memory *remediation*-oriented techniques developed in the last decade; VR, CBCR, and NBS.

## Methods

### Search method and article selection

A systematic literature search was performed using PubMed and Web of Science for studies published between January 2004 and August 2014 using the terms *Acquired Brain Injury, (Traumatic) Brain Injury* or *Stroke* in combination with *Virtual Reality, Computer-based Cognitive Retraining, Computerized Training, Non-Invasive Brain Stimulation, Transcranial Magnetic Stimulation, repetitive Transcranial Magnetic Stimulation*, or *transcranial Direct Current Stimulation* as well as *Memory*. The search in PubMed was limited in the following features: publication date (published in the last 10 years), species (human), adults (≥19 years of age), and language (English). Likewise, the search in Web of Science was limited in the following features: language (English) and time span (2004 to 2014).

Intervention studies for improving memory function after ABI were selected when they met the following inclusion criteria: (1) individuals experiencing memory deficits resulting from ABI as confirmed by neurological examination; (2) ≥18 years of age; (3) studies using specific measurements of memory functioning consisting of objective measures of memory function using standardized memory tests or batteries; and (4) had a study design with at least a pre and post intervention measurement. Memory treatment was considered any cognitive intervention attempting to improve memory, with neuropsychological tests as outcome measures. Studies published in languages other than English were excluded.

The first author (L.A.S.) conducted the search and screened the titles and abstracts, followed by an exclusion of duplicates. From screen-positive abstracts, full-text articles were collected when available and evaluated. In case the first author had doubt about inclusion of an article, the other authors were consulted. Articles meeting the aforesaid criteria were included in the final selection. The final selection was checked by the other authors as well.

### Data extraction

After the final selection, data extraction was performed by the first author (L.A.S.) and was based on data extraction methods from similar reviews (Schrijnemaekers et al., 2013). In case of doubt about which data to be extracted, the other authors were consulted. The following *study characteristics* were extracted from the articles: authors, design of the study, number of patients, outcome measures, *p*-value, and timing of measurements. The following *intervention characteristics* were extracted from the articles: aim of intervention, type of intervention, duration and intensity. The following *patient characteristics* were extracted from the articles: diagnostic criteria and severity, age, and time after onset. Results were considered to be positive when statistically significant at the *P* < 0.05 level.

### Quality assessment

Two authors (L.A.S. and T.C.W.N.) independently appraised the characteristics and the quality of the selected studies. The methodological quality was evaluated based on the following elements: (1) randomization of intervention or different condition, (2) comparison of an experimental group and a control group that received either an alternative form of treatment or no memory intervention, (3) blinding of participants, (4) blinding of researchers, (5) reporting completeness of follow-up (Tijssen and Assendelft, 2003). We added 3 relevant elements to evaluate the methodological quality: (6) group size (≥10 per group), (7) reporting effect size, and (8) reporting time post-ABI. We consider it important to report the time between the injury onset and the start of the intervention, to facilitate a comparison of the effect and to gain insights into the phase in which the patients were at time of the intervention (sub-acute phase vs. chronic phase). This 8-point checklist yielded a total score between 0 and 8. Each study were giving a total score and consequently divided into high (total scores ≥6), moderate (≥3 and ≤6), and low (≤3) quality studies (Schrijnemaekers et al., 2013). Additionally, a distinction was made between effectiveness studies and feasibility studies for the interpretation of the results of each study.

## Results

**Figure 1:**
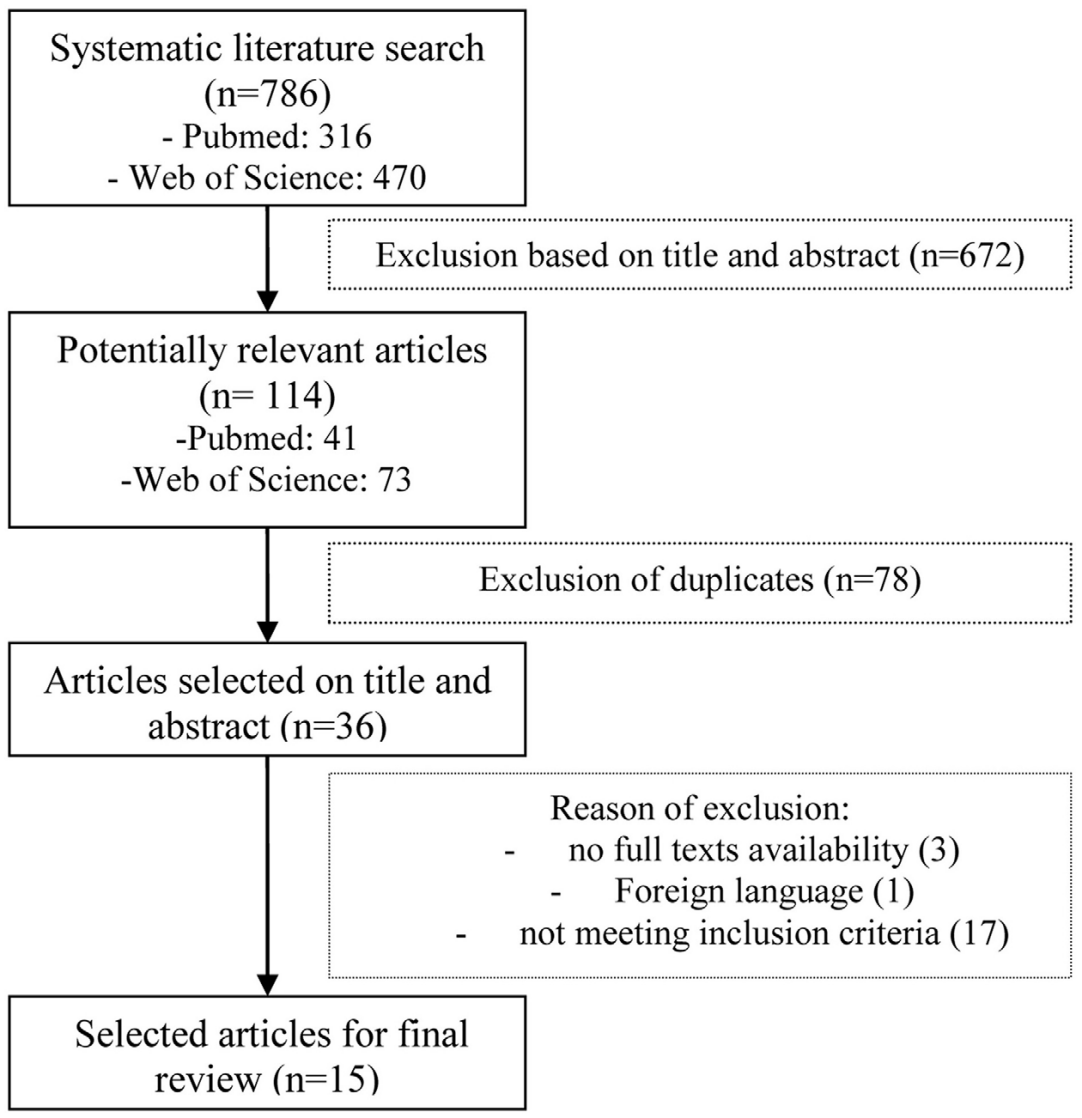
**Flowchart of the selection of article**.

The initial search identified 786 articles that were evaluated according to the inclusion criteria. Ultimately, 15 articles met the full inclusion criteria and were used for this review (see Figure 1). Of these 15 studies, 3 studies represented the VR technique, 7 studies represented CBCR, and 5 studies NBS. The specifics of the selected studies are presented in Tables 1–3 for results on VR, CBCR, and NBS respectively. After briefly describing these studies, we present the findings of the methodological quality based on the elements mentioned above (for total overview see Table 4). There was a 95% agreement between the 2 authors (L.A.S. and T.C.W.N.) regarding the quality assessment.

**Table 1:** **Virtual Reality (VR) Overview study, intervention, patient characteristics**.

**Study characteristics**						**Intervention characteristics**			**Patient characteristics**		
**Authors**	**Design**	**Number of patients**	**Outcome measures**	***p*-value**	**Measurements *Follow-up***	**Aim**	**Intervention**	**Duration/Intensity**	**Diagnostic Criteria**	**Mean age**	**Time after onset**
Caglio et al., 2012	Case-study	1	CBTT		Pre, Post 2 m, 1 y	Examining effect of navigational training using 3D VR-based video game on spatial and verbal memory	3D VR-based Videogame. Effective in activating hippocampal formation improving spatial and verbal memory.	90-min session/3 times a wk	TBI moderate	24	1 y
			CST	IR:0.03^*^				5 wk total			
				DR:0.05^*^							
			Digit Span	NS							
			RAVLT	IR:0.05^*^							
				DR: NS							
			TMT	NS							
			Phonemic Fluency	NS							
			ADAS	Improved but NS							
			RBMT	Improved but NS							
			fMRI	More extended activation							
Yip and Man, 2009	Small sample Pre-post experimental design	4	Built-in parameters	Improved but NS (all patient)	Pre Post	Test the usability and effect of newly-developed VR-based training on memory in ABI.	VR-based community living skills training program. Cognitive training elements (including memory)	35–40 min session/3 times a wk	3 stroke 1 TBI	Not specified	8 to 30 m
			NCSE Memory	Improved but NS (3/4)				10 sessions total			
			Lawton IADL	Improved but NS (3/4)							
			Self-efficacy questionnaire	Improved but NS (3/4)							
Yip and Man, 2013	Single-blind, pre-post test randomized controlled experimental design	37	Built-in Parameter:	<0.05^*^	Pre Post	Investigate the effect of VR-based PM training program on PM function for ABI patients.	VR-based Prospective memory (PM) training program. Using every day PM activities as training content.	30–45 min session/2 times a wk	Not specified	E: 37.8	E: 145.13 d
		(E: 19)	IR	NS						SD: 10.58	SD: 97.46
		(C: 18)	DR	<0.001^****^				12 sessions total		C: 38.5	C: 167.53 d
			Event	<0.001^****^						SD: 11.4	SD: 149.40
			Time	<0.001^****^							
			Time checks								
			CAM PROMT-CV	<0.05^*^							
			HKLLT	NS							
			FAB	<0.01^***^							
			WFT-CV	<0.01^***^							
			CTT	NS							
			BC-PM:	<0.01^**^							
			Event	<0.01^**^							
			Time	NS							
			Ongoing task								
			CIQ	NS							
			Self-efficacy questionnaire	<0.01^**^							

*Abbreviations: y, Years; m, months; wk, week; d, days; h, hours; min, minute; s, seconds; ES, Effect size; SD, Standard Deviation*.

NS, Not Significant; significant level p < 0.05, ^*^ significant level p < 0.01^**^, significant level p < 0.005^***^, significant level p < 0.001^****^.

*E, Experimental group; C, Control group*.

*Abbreviations neuropsychological tests: CBTT, Corsi Block Tapping Test; CST, Corsi's Supraspan Test; RAVLT, Rey Auditory Verbal, Learning Test; IR, Immediate Recall; DR, Delayed Recall; TMT, Trial Making Test; ADAS, Alzheimer's Disease assessment Scale; RBMT, Riverhead Behavioral Memory Test; fMRI, Functional Neuroimaging assessment; NCSE, The Neurobehavioral Cognitive Status; Lawton IADL, Lawton Instrumental Activities of Daily Living Scale; MMSE-CV, Chinese Version Mini Mental State Examination; TONI-3, Test of Nonverbal Intelligence – 3rd Edition; SADI-CV, Chinese Version of the Self-Awareness of Deficit Interview; CAMPROMT-CV, The Cambridge Prospective Memory Test Chinese Version; HKLLT, Hong Kong List Learning Test; FAB, Frontal Assessment Battery; WFT-CV, Word Fluency Test; CTT, Color Trail Test; BC-PM, Behavioral Checklist of prospective Memory Task in a real environment; CIQ-CV, Chinese version of the Community Integration Questionnaire*.

**Table 2:** **Computer-Based Cognitive Retraining (CBCR) Overview study, intervention, patient characteristics**.

**Study characteristics**						**Intervention characteristics**			**Patient characteristics**		
**Authors**	**Design**	**Number of patient**	**Outcome measures**	***p*-value**	**Measurements *Follow-up***	**Aim**	**Intervention**	**Duration/Intensity**	**Diagnostic criteria**	**Mean age**	**Time after onset**
Westerberg et al., 2007	Randomized pilot design	18	Span Board	<0.05^*^	Pre Post	The effect of intense, adaptive WM training on various visuo-spatial, auditory modalities after stroke.	Computer-based visuo-spatial and auditory working memory training. (Cogmed QM)	40-min session	Stroke (mild to severe)	54	12–36 m
		(E: 9)		ES.83				5 times a wk (90 trials a d)		SD: 7.7	
		(C: 9)						5 wks total			
			Digit-Span	<0.005^***^							
				ES: 1.54							
			Stroop	NS							
				ES.24							
			Claeson Dahl	NS							
			Raven's PM	NS							
				ES.1							
			Word list:	NS ES.3							
			Repetitions	NS ES.05							
			DR								
			PASAT	<0.001^****^							
				ES.61							
			RUFF	<0.005^***^							
				(ES + 0.81)							
			CFQ	<0.005^***^							
				ES.8							
Li et al., 2013	Quasi-experimental pre-post test design	11	Cognistat Assessment:		Pre Post	CBCR program on improving memory and attention in ABI patients.	CBCR Parrot Software: 8 subprograms for attention and memory.	60-min session/10 lessons per session/1 session a wk	Not specified	49.45	21.27
			Attention	<0.005^***^				8 sessions total.		SD: 19.02	SD: 14.21
			Memory	<0.05^*^							
Lundqvist et al., 2010	Controlled cross-over experimental design	21	PASAT:	<0.001^****^	Pre (A1), Post (A2), 20 wks (A3)	Examining the short- and long-term effects of CBCR on visuo-spatial and verbal WM.	Computer-based training visuo-spatial and verbal WM tasks (Cogmed QM)	45–60 min session/5 times a wk	1 TBI	43.3	46.4
		(E: 10)	A1-A2	<0.001^****^				5 wks total	11 Stroke	SD: 9.8	SD: 31.9
		(C: 11)	A1-A3						5 Infection		
									2 Tumor		
									2 SAH		
			CWIT:	<0.001^****^							
			A1-A2	NS							
			A1-A3								
			Block-Span	NS							
			LST:	<0.001^****^							
			A1-A2	<0.001^****^							
			A1-A3								
			Picture Span	NS							
			COPM:	<0.05^*^							
			A1-A3								
			EQ-5D	NS							
			VAS	NS							
Johansson and Tornmalm, 2012	Prospective cohort design	18	Built-in Parameter	0.000^****^	During Post 6 m	Effect of computer-based program on daily WM functioning in ABI.	Working Memory	30–45 min session/3 times a wk	5 TBI	47.5	7 y
							Training Program (Cogmed QM)	7–8 wks total	6 Tumor	SD: 13	SD: 6.35
									7 Stroke		
			CFQ	0.018^*^							
			COPM:	0.008^***^							
			Perf. Satify.	0.01^**^							
Björkdahl et al., 2013	Randomized controlled design	38	Digit B:	0.003^***^	Pre (A1), Post (A2), 18 wks (A3) C: 24 wks (A4)	Explore feasibility and effect of computer-based program on memory function after ABI.	Working memory training program (Cogmed QM)	30–45 min session/5 d a wk	28 stroke	51	27 wks
		(E: 20)	A1-A2	ES – 0.48				5 wks total.	5 TBI	SD: 11	
		(C:18)	A1-A3	0.000^****^					5 Other		
				ES-0.56							
			AMPS:	0.016^*^							
			A1-A3	ES -0.38							
			A2-A3	0.038^*^							
				ES - 0.33							
			RBMT II	0.042^***^							
			A3-A4	(ES - 0.51)							
			FIS:	0.038^*^							
			A1-A2	ES – 0.33							
			WM Q:	0.006^***^							
			A1-A3	ES – 0.44							
De Luca et al., 2014	Control design	35	Levels	<0.001^****^	Pre (A1)	Evaluate the effect of cognitive	Cognitive PC-training: memory, executive functions, abilities of thinking.	24 sessions, 3 times a wk.	TBI 48.57%	E: 30.93	Not specified
		(E:15)	Cognitive	0.004^***^	Post (A2)	PC-training in ABI patients.			Stroke	SD: 11.10	
		(C:20)	Functioning		2 m (A3)					C: 39.75	
			A1-A2								
			A1-A3							SD:15.43.	
			RML	0.009^**^							
			A1-A2	0.002^***^							
			A1-A3								
			Attentive	0.008^**^							
			Matrices	<0.001^****^							
			Matrices	<0.001^****^							
			A1-A2								
			A1-A3								
			MMSE	0.002^**^							
			A1-A2	0.002^**^							
			CVF	0.002^***^							
			A1-A2	0.03^*^							
			A1-A3								
			LVF	0.007^**^							
			A1-A2	<0.001^****^							
			A1-A3								
			RAVLT IR	0.001^**^							
			A1-A2	<0.001^****^							
			A1-A3								
			RAVLT Recall	0.009^**^							
			A1-A2	0.007^**^							
			A1-A3								
			Constructional	0.03^*^							
			Apraxia	0.002^**^							
			A1-A2								
			A1-A3								
			Activities	0.005^**^							
			Daily Living	0.005^**^							
			A1-A2	0.02^*^							
			A1-A3								
			IADL	0.005^**^							
			A1-A2	0.02^*^							
			A1-A3								
			Barthel Index	0.002^**^							
			A1-A2	0.07							
			A1-A3								
			Hamilton	0.94							
			Anxiety	0.01^*^							
			A1-A2								
			A1-A3								
			Hamilton	0.009^**^							
			Depression	0.009^**^							
			A1-A2								
			A1-A3								
Fasotti and van Kessel, 2013	Randomized study	45	Digit F	0.003^***^	Pre (A1), 6 wks (A2), 18 wks (A3)	Explore the effect of WM-training on WM, cognition, physiological health in ABI patients	Working memory training program (Cogmed QM)	30–45 min session/5 d a wk	E: 17 Stroke	E: Median: 51	E: Median: 32 wks
		(E: 25)	A1-A2	0.002^***^				5 wks total	4 TBI	Range: 22–63	Range: 12–135
		(C: 20)	A1-A3						4 Other	C: Median: 53	C: Median 22.5 wks
									C: 15 Stroke	Range: 22–62	Range: 16–500
									3 TBI		
									2 Other		
			Digit B	0.004^***^							
			A1-A2	0.0001^****^							
			A1-A3								
			Span board F	NS							
			Span Board B	NS							
			Span Board	NS							
			Scaled Score								
			WAIS III WM scale	0.000^****^							
			A1-A2	0.003^***^							
			A1-A3								
			BNIS	0.006^***^							
			A1-A2	0.006^***^							
			A1-A3								
			DEX	NS							

*Abbreviations: y, Years; m, months; wk, week; d, days; h, hours; min, minute; s, seconds; ES, Effect size; SD, Standard Deviation*.

NS, Not Significant; significant level p < 0.05, ^*^ significant level p < 0.01^**^, significant level p < 0.005 ^***^, significant level p < 0.001^****^.

*E, Experimental group; C, Control group*.

*Abbreviations neuropsychological tests: Stroop, Stroop Interference Test; Raven's PM, Raven's Progressive Matrices; CFQ, Cognitive Failure Questionnaire; PASAT, Paced Auditory Serial Addition Test; RUFF, RUFF 2 & 7 Selective Attention Test; Cogmed, Cogmed Cognitive Medical System; CWIT, Color Word Interference Test Condition 4-Inhibition/Switching; SAH, Subarachnoidal Hemorrhage; Block-Span, Block-Span Board Forward and Backwards from WAIS R-NI; LST, Listening Span Task; Picture Span, The Picture Span; COPM, Canadian Occupational Performance Measure; EQ-5D, EQ-5D Questionnaire; VAS, Health Self-Rating VAS; AMPS, Assessment of Motor and Process skills; RBMT II, Riverhead Behavioral Memory Test II; FIS, Fatique Impact Scale; WMQ, Working Memory Questionnaire; RML, Revearsal Motor Learning; CVF, Category Verbal Fluency; LVF, Letter Verbal Fluency; RAVLT, Rey Auditory Verbal, Learning Test; IR, Immediate Recall; DR, Delayed Recall; IADLC, Instrumental Activities of Daily Living Scale*.

**Table 3:** **Non-Invasive Brain Stimulation (NBS) Overview study, intervention, patient characteristics**.

**Study characteristics**						**Intervention characteristics**			**Patient characteristics**		
**Authors**	**Design**	**Number of patients**	**Outcome measures**	***p*-value pre vs. post**	**Measurements *Follow-up***	**Aim**	**Intervention**	**Duration/Intensity**	**Diagnostic criteria**	**Mean age**	**Time after onset**
Kim et al., 2010	Single-center, prospective, double-blind, sham-controlled preliminary design	18	Digit F	NS	Pre Post	Effect of rTMS over left DLPFC on cognition and mood in post-stroke patients	Group 1 1-Hz stimulation at 80% MT.	G1: 3 trains of 5 min duration each, separated by 1 min pauses. Total period of 20 min (a total of 900 pulses).	18 Stroke	Group 1 63.3	Group 1 404.4 d
		(1 Hz: 6)	Digit B	NS			Group 2 10-Hz stimulation at 80% MT.	G2: 3 blocks, separated by 10-min interval.		SD: 7.4	SD 71.7
		(10 Hz: 6)	Forward visual span	NS			Control group Sham stimulation was delivered as 1 HZ protocol except the angle of the coil was at 90% perpendicular to the skull.	Each block: 15 trains of 1 s duration separated by 10 s pauses (a total of 450 pulses).		Group 2 53.5	Group 2 241.2 d
		(C: 6)	Backward visual span	NS						SD: 16.9	SD: 42.5
			Verbal learning test	NS						Control 66.8	Control 69.7 d
			Visual learning test	NS						SD: 17.2	SD: 39.0
			Auditory CPT	NS							
			Visual CPT	NS							
			Word of color word test	NS							
			Color of color word test	NS							
			Tower of London test	NS							
			MBI	NS							
			BDI	NS							
Jo et al., 2009	Single-blind cross-over and sham-controlled design	10	2-back verbal		Pre Post	Effect of Anodal tDCS over left DLPFC on WM after stroke.	tDCS constant current of 2 mA over DLPFC. 48 h interval. Sham stimulation.	30-min session/two conditions: Anodal /sham stimulation.	10 Stroke	47.9	Not specified
			WM task:							SD: 8.7	
			Accuracy	<0.05^*^							
			Recognition	<0.05^*^							
			Response time	NS							
Ulam et al., 2014	Randomized double-blind design	26	Elevator count w distraction	0.945	Pre Post	Effect of anodal tDCS over DLPFC on attention and WM on EEG oscillations and neuropsychological tests in TBI.	tDCS constant current of 1 mA anodal on DLPFC	20-min session/10 days/One session a d.	26 TBI	Active: 31.34	Active: 57.38 d
		(E: 13)								SD: 9.8	SD: 37.8
		(C: 13)	Visual elevator accuracy	0.003^***^						Sham: 35.70	Sham: 41.08
										SD: 14.7	SD: 20.87
			Visual elevator time	0.035^*^							
			Elevator count w reversal	0.008^**^							
			Digit F	0.71							
			Digit B	0.034^*^							
			Digit S	0.0001^****^							
			Symbol span	0.0001^****^							
			Color naming time	0.002^***^							
			Word reading time	0.011^*^							
			Inhibition time	0.001^****^							
			Inhibition accuracy	0.0001^****^							
			Inhibit/switch time	0.004^***^							
			Inhibit/switch accuracy	0.001^****^							
			TASIT	0.0002^****^							
			HVLT total recall	0.26							
			HVLT delayed recall	0.06							
			BVMT total recall	0.006^**^							
			BVMT delayed recall	0.03^*^							
Leśniak et al., 2014	Randomized, sham-control, double blind pilot design	23	RAVLT	0.1 (ES.36)	3 wk before, Pre Post 4 m	Effect of repeated application of anodal tDCS would enhance the effects of specific cognitive training to improve memory and attention in TBI.	Cognitive computerized training: 15 sessions. Content: internal strategies. Prior to each session, the patient received either active or sham anodal tDCS.(1 mA over DLPFC)	Active: 15 sessions of anodal tDCS (1 mA)/10 min/current density = 0.028 mA/cm^2^. Control: 15 sessions of sham anodal tDCS (1 mA for 25 s).	23 TBI	Active: 28.3	Active: median: 10.8 m range: 5.8–18.5
		(E:12)	Learning							SD: 9	Sham: median 13.2 m range: 6.5–25.1
		(C:11)								Sham: 29.3	
										SD: 7.7	
			RAVLT	Sham.93							
			Delayed recall	(ES.02)							
				tDCS.21							
				(ES.26)							
			RAVL	Sham.95						
			Delayed recognition	(ES.01)						
				tDCS.21						
				(ES.26)						
			PRM	Sham.07							
			Immediate recall	(ES.38)							
				tDCS.23							
				(ES.25)							
			PRM	Sham.18							
			Delayed Recognition	(ES.29)							
				tDCS.23							
				(ES.25)							
			PASAT	0.06							
				(ES.31)							
			SSP	Sham.27							
				(ES.23)							
				tDCS.03^*^							
				(ES.43)							
			RVP	0.007^**^							
				(ES.66)							
			EBIQ	0.41							
Park et al., 2013	Double-blind, sham-controlled pilot design	11	SCNT		Pre Post	Effect of anodal tDCS combined with CACR to improve cognitive function.	CACR: 15-min memory training and 15-min attention training. 30 min a d/5 d a wk/until discharge	Active Anodal tDCS over bilateral PFC (2 mA) for 30 min. Control: tDCS over bilateral PFC (2 mA) for 30 s.	11 Stroke	Active: 65.3	Active: 29.0 d
		(E: 6)									
		(C: 5)	Digit F	0.429						SD: 14.3	SD: 18.7
			Digit B	0.931						Sham: 66.0	Sham: 25.2 d
			Visual span forward	0.931						SD: 19.8	SD: 17.5
			Visual span backward	0.931							
			Auditory CPT	0.017^*^							
			Auditory controlled CPT	0.792							
			Visual CPT	0.017^*^							
			Visual controlled CPT	0.792							
			MMSE	0.931							

*Abbreviations: y, Years; m, months; wk, week; d, days; h, hours; min, minute; s, seconds; ES, Effect Size; SD, Standard Deviation*.

NS, Not Significant; significant level p < 0.05, ^*^ significant level p < 0.01^**^, significant level p < 0.005 ^***^, significant level p < 0.001^****^.

*E, Experimental group; C, Control group; LFS, Low frequency Stimulation; DLPFC, Dorsolateral Prefrontal Cortex; MT, Motor Threshold*.

*Abbreviations neuropsychological tests: BDI, Beck Depression Inventory; CPT, Continuous Performance Test; MDI, Modified Barthel Index; Verbal ALT, Verbal Associate Learning Test; Digit F, Digit Span Forward; Digit B, Digit Span backward; Digit S, Digit span sequencing; Visual ALT, Visual Associate Learning Test; VALT Tap F, Visual Associate Learning Test Taping forward; VALT Tap B, Visual Associate Learning Test Taping Backward; VCPT, Vigilance Continuous Performance Test; MMSE, Mini-Mental Status Examination; HVLT, Hopkins Verbal Learning Test; BVMT, Brief Visual Memory Test; RAVLT, Rey's Auditory Verbal Learning Test; PRM, Pattern Recognition Memory Test; PASAT, Paced Auditory Serial Addition Test; SSP, Spatial Span Test; RVP, Rapid Visual Processing*.

**Table 4:** **Scores of the quality assessment of the discussed studies, based on 8 elements**.

**Study**	**1**	**2**	**3**	**4**	**5**	**6**	**7**	**8**	**Total score**	**Quality**	**Aim**
**VR**											
Caglio et al., 2012	0	0	0	0	1	0	0	1	**2**	**Low**	Feasibility
Yip and Man, 2009	0	0	0	0	1	0	0	1	**2**	**Low**	Feasibility
Yip and Man, 2013	1	1	0	1	1	1	0	1	**6**	**High**	Effectiveness
**CBCR**											
Westerberg et al., 2007	1	1	1	1	1	0	1	1	**7**	**High**	Effectiveness
Li et al., 2013	0	0	0	0	0	1	0	1	**2**	**Low**	Effectiveness
Lundqvist et al., 2010	1	1	0	0	1	1	1	1	**6**	**High**	Effectiveness
Johansson and Tornmalm, 2012	0	0	0	0	1	1	0	1	**3**	**Moderate**	Feasibility
Björkdahl et al., 2013	1	1	0	0	1	1	1	1	**6**	**High**	Effectiveness
De Luca et al., 2014	1	1	0	0	1	1	0	1	**5**	**Moderate**	Effectiveness
Åkerlund et al., 2013	1	1	0	0	1	1	0	1	**5**	**Moderate**	Effectiveness
**NBS**											
Kim et al., 2010	1	1	1	1	0	0	0	1	**5**	**Moderate**	Effectiveness
Jo et al., 2009	1	0	1	0	1	1	0	0	**4**	**Moderate**	Effectiveness
Ulam et al., 2014	1	1	1	1	1	1	1	0	**7**	**High**	Effectiveness
Leśniak et al., 2014	0	1	1	1	1	1	1	1	**7**	**High**	Effectiveness
Park et al., 2013	1	1	1	1	0	0	0	1	**5**	**Moderate**	Effectiveness

*0, Negative; 1, Positive*.

*High was considered total scores ≥ 6, moderate ≥ 3 and ≤ 6, and low ≤ 3*.

*Elements: 1, Randomization of intervention or different condition; 2, Comparison of an experimental group and a control group; 3, Blinding of participants; 4, Blinding of researchers; 5, Reporting completeness of follow-up; 6, Group size ≥ 10 per group; 7, Reporting Effect Size; 8, Reporting time post-ABI*.

### Virtual Reality

A small-sample (*n* = 4), pre and post experimental design was developed to initially study the usability and efficacy of a VR-based training program for people with ABI (Yip and Man, 2009). Outcome measures were at the level of memory function. A VR-based community living skills training of 10 sessions were given, consisting of key cognitive training elements (including memory) to promote generalization to real-life situations. Measurements consisted of built-in parameters to document the participants' performance during each session and the Neurobehavioral Cognitive Status Examination. Due to the explorative and qualitative character of this study, a statistical significance was not established. However, a positive training effect was shown by the outcome measures and narratively presented. All 4 patients showed improvement in skills acquisition on the community living tasks and in memory performance on neuropsychological measurements. All patients showed the same improvement in performing the tasks when tested again in a real-life environment.

Caglio et al. (2012) described a qualitative case-study of a 24-year-old man with TBI presenting memory deficits and evaluated the efficacy of a 3D interactive VR navigational training program measuring neuropsychological changes and fMRI modification cerebral activations. Measurements consisted of a functional neuroimaging assessment and a standardized neuropsychological assessment on frontal executive functions, general cognitive functions, and various memory functions (i.e., spatial short-term memory, visual-spatial learning, WM, verbal learning). Visual-spatial memory improvement appeared to be present both after the VR navigational training and in follow-up testing. The functional neuroimaging assessment showed an increased cerebral activity in the left hippocampus and the right parahippocampal cortex compared to the pre-training assessment.

Four years later, Yip and Man described the effectiveness of a VR-based memory training in a larger sample (*n* = 37) (2013). Using a *Randomized Controlled Trial* (RCT), the effectiveness was evaluated of a VR-based cognitive rehabilitation program using prospective memory as training content. While the experimental group received a 12-session VR-based program, the control group did not received any VR-based training, but did attend regular readings and table game activities during the treatment phase. Neuropsychological tests were administered to measure the effects of the treatment on prospective memory skill acquisition (VR-based assessment by outcome parameters), prospective memory, learning, and executive function. The results showed significantly larger changes in both VR-based and real-life prospective memory outcome measures after the training. Related cognitive attributes such as frontal lobe functions and semantic fluency showed a significant improvement compared with the control group. A significant improvement is found in the real-life behavioral assessment in prospective memory due to the training, indicating a transfer of learnt skills in a virtual environment to a real-life setting.

The study of Yip and Man (2013) was considered to be of high quality. Both the studies of Caglio et al. (2012) and Yip and Man (2009) were considered to be of low quality based on the quality assessment. It should be noted however that a true comparison was difficult to make as 2 studies were qualitative research (Yip and Man, 2009; Caglio et al., 2012) whereas 1 was quantitative research (Yip and Man, 2013).

To sum up, although the 3 studies do identify an improvement in memory function after VR-based training, it is difficult to draw any conclusions as the number of articles available was limited. Besides, of all the available articles available only 1 study was considered of high quality.

### Computer-Based Cognitive Retraining

Using a RCT, the effects of intense adaptive WM training in stroke patients were investigated (Westerberg et al., 2007). A sample of 18 patients was randomly divided into an experimental or passive control group. The experimental group was trained using computer-based visuo-spatial and auditory WM tasks at home. The training method was implemented with the software program Cogmed QM (Cognitive Medical Systems). The control group only performed the neuropsychological test battery with no training in between at baseline and after 5 weeks. Both WM and attention abilities improved significantly within the experimental group, but not within the passive control group.

In a cross-over RCT, the short- and long-term transfer effects of a computerized WM training program were evaluated for patients suffering of WM deficits after ABI (Lundqvist et al., 2010). A sample of 21 patients was randomly divided into 2 groups. The experimental group received systematic WM training (Cogmed QM), whereas the control group did not receive any training during the same period. The patients were assessed at baseline, after 4 and 20 weeks with neuropsychological tests focusing on verbal and visual WM. There was a significant improvement on the trained WM tasks and the non-trained WM tasks as measured by neuropsychological tasks at 4 and 20 weeks after training compared to baseline.

A prospective cohort study evaluated the effectiveness of a computerized training using Cogmed QM software (Johansson and Tornmalm, 2012). A sample of 18 ABI patients attended the training 3 times a week. The patients were assessed before, during, after the WM training, and additionally at a 6 months follow-up with WM assessments. The computerized training showed a significant improvement on trained WM tasks. The effect was maintained at the 6 month follow-up. The study supports the idea that a computerized WM training program can affect WM functioning for ABI patients.

An additional RCT assessed the effectiveness of computerized WM training (Cogmed QM) on WM functioning in ABI patients (Björkdahl et al., 2013). A sample of 38 ABI patients were randomly assigned to an experimental group or control group and received 5 weeks of standard rehabilitation in accordance with the usual routine at the clinic. The experimental group was offered an additional training with the Cogmed QM training program. To explore the impact of the training, assessments were done at baseline, after the training program, and at a follow-up 3 months later. The assessment consisted of neuropsychological tests and a WM questionnaire measuring WM on functional level (i.e., level of activity in daily life). The experimental group improved significantly more compared to the control group. Cogmed QM showed a generalized effect on non-trained WM tasks.

Using a quasi-experimental pre-test and post-test design the effectiveness of a CBCR program was evaluated on improving memory and attention deficits for adults with ABI (Li et al., 2013). A sample of 12 patients was assessed using the Cognistat Assessment as pre-test and post-test measurement. Each patient completed 8 sessions using the attention and memory subprograms of the Parrot Software, which is an interactive rehabilitation program with over 100 subprograms designed to improve cognitive function. Significant improvement was found in both memory and attention measured by the Cognistat Assessment scores.

A RCT investigated whether patients with a dysfunctional WM could improve their WM, cognitive function, and psychological health using a computerized WM training with the Cogmed QM program (Åkerlund et al., 2013). A sample of 47 patients, in the sub-acute phase after ABI, were randomly assigned into an intervention group and a control group. Various WM neuropsychological tests were administered at baseline, post-intervention, and at a follow-up of 18 weeks. Both groups underwent integrated rehabilitation. The intervention group also attended the computerized WM training program, which was offered to the control group after the completion of the study. Both the Barrow Neurological Institute Screen for Higher Cerebral Functions (BNIS) and the Digit Span differed significantly between the intervention and control group due to the greater improvement in the intervention group after the WM training. Both groups improved after WM training on the BNIS, the Digit Span, and the WAIS III WM scale. Additionally, psychological health improved as both groups reported less depressive symptoms.

A RCT evaluated the effects of a cognitive pc-training with regard to semantic memory, verbal fluency, and short-term auditory-verbal memory in ABI patients (De Luca et al., 2014). A sample of 35 ABI patients were randomly divided into 2 groups. Cognitive impairment was investigated through the use of a psychometric battery, administered before and 2 months after the cognitive pc-training. The cognitive pc-training was performed only by the experimental group, in addition to conventional treatment. After the training, the results showed a global improvement in both of the groups. However, the experimental group showed a greater cognitive improvement than the control group, with significant differences in all the neuropsychological tests performed. The results suggest that cognitive pc-training may be a promising methodology to optimize rehabilitation outcomes following ABI.

Based on the evaluated elements from the quality assessment, the studies of Westerberg et al. (2007), Lundqvist et al. (2010), and Björkdahl et al. (2013) were considered of high quality (see Table 4). The studies of Johansson and Tornmalm (2012), De Luca et al. (2014), and Åkerlund et al. (2013) were considered of moderate quality. The study of Li et al. (2013) was considered of low quality.

To sum up, the 7 studies reported a significant improvement of the memory function after the completion of CBCR. As 6 studies were considered of moderate/high quality, these findings support the idea that CBCR may be a promising methodology to optimize the recovery of the memory function in ABI patients.

### Non-Invasive Brain Stimulation

A single-blind, cross-over, and sham-controlled study investigated whether anodal tDCS over the left dosolateral prefrontal cortex would affect the WM performance of post-stroke patients (Jo et al., 2009). A sample of 10 patients participated in 2 stimulation conditions (anodal stimulation with a constant current of 2 mA and sham stimulation). The order of stimulation was randomly assigned. Each stimulation session was separated by at least 48 h to wash out the effects of the previous run. All patients performed a two-back WM task before and after the administration of the tDCS. A significant improvement in accuracy and recognition accuracy was only found in the anodal tDCS and not in the sham tDCS. Anodal tDCS applied over the left dorsolateral prefrontal cortex at an intensity of 2 mA was associated with enhanced verbal WM performance in patients after stroke.

A double-blind RCT examined whether rTMS applied over the left dorsolateral prefrontal cortex affected cognition and mood in post-stroke patients (Kim et al., 2010). A sample of 18 patients were enrolled and randomly assigned to 1 of 3 treatment groups: low-frequency (1 Hz) stimulation, high-frequency (10 Hz) stimulation, and sham stimulation (control). Each patient underwent 10 consecutive treatment sessions (5 times a week for 2 weeks). A complete neuropsychological battery was performed to evaluate various domains of cognition such as verbal and visual memory, executive functioning, attention, working memory, and visuo-motor coordination. The Beck Depression Inventory was used to assess mood status. These assessments were conducted in all patients before and after treatment. Treatment had no significant effect on any cognitive function parameter in any of the 3 groups. In contrast, high-frequency rTMS resulted in significantly lower Beck Depression Inventory scores compared with baseline, and compared with the other 2 groups. These preliminary data suggests that there was a positive effect on mood, but the study was not powered to detect any measurable effect on cognition including memory.

A double-blind RCT investigated the synergistic effects of both computer-assisted cognitive rehabilitation (CACR) and tDCS on cognitive function (attention and memory) in post-stroke patients (Park et al., 2013). A total of 11 patients were randomly divided into an active tDCS group and a control group. Both groups received CACR training for 30 min a day (15 min memory training, 15 min attention training) 5 times a week until discharge. The tDCS group completed the CACR program during a mean period of 18.5 days combined with the anodal tDCS (over the bilateral prefrontal cortex). The control group also completed the CACR program (mean period of 17.8 days) combined with tDCS, except that the current was reduced to 0 after 30 s. All patients were evaluated using the Korean Mini-Mental State Examination and the Seoul Computerized Neuropsychological Test (SCNT). The SCNT was composed of 10 measurements, assessing the verbal memory, visuospatial memory, attention, and visuo-motor coordination. The patients of the tDCS group showed a significant improvement in 2 attention tests of the SCNT items. The results indicated that the combined use of tDCS and a CACR program may provide beneficial effects in improving attention. However, no evidence was found for the memory function.

A double-blind RCT investigated the cumulative effects of anodal tDCS on EEG oscillations, attention, and WM function among patients with TBI (Ulam et al., 2014). A sample of 26 patients were randomly assigned to active or sham tDCS groups. EEGs were recorded at 6 different time points, assessing both immediate and cumulative effects of tDCS on EEG oscillations. Twenty-minutes sessions of 1 mA anodal tDCS over the left dorsolateral prefrontal cortex were provided on 10 consecutive days for the active group. For the sham tDCS group, current gradually faded in over a period of 8 s, followed by 30 s of stimulation, with the current then fading out over an additional 8 s. Neuropsychological tests were administered before and after the series of tDCS sessions. While attention and WM was the primary interest, other outcome measures were included. Results show that no between-group differences were present for any of the tests administered. Both the active tDCS and sham tDCS showed an equal number of statistically significant improvements (15 out of 19 tests). The EEG revealed immediate and cumulative changes in brain oscillations for the active tDCS, but not in the sham group. Results suggest that 10 anodal tDCS sessions may beneficially modulate regulation of cortical excitability for patients with TBI. However, tDCS does not show greater improvements on neuropsychological test (including measurements of WM and attention) compared to sham tDCS.

A double-blind RCT determined whether cumulative anodal tDCS over the left dorsolateral prefrontal cortex could enhance rehabilitation of memory and attention in patients with TBI (Leśniak et al., 2014). A sample of 23 patients were randomly assigned to 2 groups. The experimental group received anodal tDCS (10 min of 1 mA) on a daily basis for 15 days followed by rehabilitative cognitive training. The control group received anodal tDCS in the first 25 s of a 10 min stimulation period (sham condition) with the same rehabilitation. A battery of memory and attention neuropsychological tests were administered which included visual and auditory modalities. Participants were tested twice before the intervention (to control for spontaneous recovery), after the intervention, and 4 months later. After treatment the experimental group exhibited larger effect sizes in 6 of 8 cognitive outcome measures, but they were not significantly different from controls. At follow-up, differences remained insignificant. This study did not provide sufficient evidence to support the efficacy of repeated anodal tDCS for enhancing rehabilitation of memory and attention in patients after severe TBI.

Based on the quality assessment the studies of Ulam et al. (2014) and Leśniak et al. (2014) were considered of high quality. The studies of Jo et al. (2009), Kim et al. (2010), and Park et al. (2013) were considered of moderate quality.

To sum up, only 1 study (Jo et al., 2009) detected a significant enhanced verbal WM performance after the use of NBS in stroke patients. Four studies (Kim et al., 2010; Park et al., 2013; Leśniak et al., 2014; Ulam et al., 2014) do not find sufficient evidence to support the efficacy of NBS for enhancing rehabilitation of memory in ABI patients.

## Discussion

The aim of this systematic review was to describe memory rehabilitation interventions based on *remediation*-oriented techniques post ABI and evaluate its effectiveness. We found 15 studies in the last 7 years (2007–2014), evaluating 3 memory *remediation* approaches; 3 studies on VR, 7 on CBCR and 5 on NBS. Considering the quality of the studies, only 1 study representing VR completed the requirements of a RCT, 3 studies representing the NBS technique fulfilled the RCT requirements, whereas 5 studies representing CBCR consisted of a RCT design. It appeared, based on the quality assessment that CBCR was the most promising as the methodological quality was high. Importantly, CBCR is found effective in improving the memory function post ABI. Although the VR studies did find a positive effect, there was only a low number of studies available and the quality of the studies was also considered low. Four of the 5 studies evaluating NBS did not find significant improvement of the memory function and the quality of these studies were considered moderate to high. Only 1 of the studies evaluating NBS did find positive results, yet the quality of this study was considered moderate. More details of the findings will be discussed below for each technique separately.

### Computer based cognitive rehabilitation

All 7 studies have shown that CBCR can be effective in improving memory function in individuals with ABI (Westerberg et al., 2007; Lundqvist et al., 2010; Johansson and Tornmalm, 2012; Åkerlund et al., 2013; Björkdahl et al., 2013; Li et al., 2013; De Luca et al., 2014). These findings are consistent with Klingberg's prediction (2010), suggesting that WM training could be used as a *remediation*-oriented intervention for individuals for whom low WM capacity is a limiting factor in everyday life.

Five studies (Westerberg et al., 2007; Lundqvist et al., 2010; Johansson and Tornmalm, 2012; Björkdahl et al., 2013; De Luca et al., 2014) investigated the generalized effect on functional level (i.e., level of activity in daily life) and found a positive effect. This was measured subjectively, using self-report questionnaires regarding daily activities relying on the memory function. This is interesting from a transfer-of-training point of view. Five studies measured the long-term effects, ranging from 18 to 24 weeks post-intervention and found positive results (Lundqvist et al., 2010; Johansson and Tornmalm, 2012; Åkerlund et al., 2013; Björkdahl et al., 2013; De Luca et al., 2014).

Five studies randomly assigned their participants to an experimental vs. a control group and therefore completed the requirements of a RCT. Only 1 study used a double blind design (both patient and researcher were blind to the treatment of the patient) (Westerberg et al., 2007). Future research should focus on the establishment of several other criteria such as blinding of participants and researchers to strengthen the evidence.

In conclusion, we consider CBCR as most promising due to the positive results and the relatively high methodological quality of the selected studies. However, before proposing CBCR as rehabilitation intervention in clinical practice important criteria should be fulfilled. Future research should further define the effect of the intervention generalized to functional level, participation in society level, as well as the long-term effects and the effect on quality of life. Additionally, RCT's are needed focusing on several methodological criteria to strengthen evidence.

### Virtual Reality

All 3 studies found a significant improvement of the memory function after a VR-based training (Yip and Man, 2009, 2013; Caglio et al., 2012). These positive findings are consistent with findings in healthy elderly participants experiencing memory deficits (Optale et al., 2009). This suggests that VR-based training could possibly be a valid part at encouraging memory recovery for individuals experiencing memory deficits.

Two studies also found a generalization of the effect in a real environment (Yip and Man, 2009, 2013). Patients showed the same improvement in performing the tasks when tested again in the real environment. As such, VR-based training seems to be able to retrain the underlying function in a virtual environment and facilitate the generalization to real-life performance.

No study investigated the effect of the training on functional level. Long term effects at 2 months and 1 year post-intervention were established in the study of Caglio et al. (2012), but should be interpreted with caution due to the low methodological quality (total score: 2). Both studies of Yip and Man (2009, 2013) have shown positive effect but for a limited time-window, namely only directly post-intervention as no follow up was performed at a longer interval post-training. In future studies, it would be desirable to extend the outcome measurements to establish the effect on functional level and the long-term effects. This could provide valuable information for clinical use.

It is important to note that the quality of the studies representing VR was low to moderate according to our quality assessment. Only 1 study (Yip and Man, 2013) met the requirements of a RCT and used a single blind design (blinding the researcher for the treatment). Two studies failed to apply a control group in their methodology. As a result, those studies did not blinded their participants nor their researchers. Future research should involve true replications, taking into account essential criteria such as randomization and the use of control group to obtain higher methodological quality.

In conclusion, despite the positive findings these results should be considered preliminary because of the limited number of studies available and the low number of ABI patients. The significant improvement on memory performance for ABI patients is encouraging. However, insufficient evidence is available to be proposed as treatment in clinical practice.

### Non-Invasive Brain Stimulation

Four studies did not detect significant improvement in the memory function after the use of NBS (Kim et al., 2010; Park et al., 2013; Leśniak et al., 2014; Ulam et al., 2014). According to the quality assessment these studies were considered moderate to high (total score ranging from 4 to 7). Only 1 study found a significant WM improvement (Jo et al., 2009), however the methodological quality of this study was considered moderate (total score: 4).

These disappointing findings were unexpected due to the promising findings in healthy participants. Several studies did find significant improvement in memory tasks due to NBS in healthy participants (Kessels et al., 2000; Oliveri et al., 2001; Luber et al., 2007; Preston et al., 2010). A recent review detected positive effects of rTMS and tDCS improving measures of WM performance, including reaction time and/or accuracy (Brunoni and Vanderhasselt, 2014). These results were only found when the NBS was applied over the dorsolateral prefrontal cortex (DLPFC) (Brunoni and Vanderhasselt, 2014). On the contrary, 3 studies of the present review did not detect significant improvement of the memory function, even when NBS was applied over DLPFC (Kim et al., 2010; Leśniak et al., 2014; Ulam et al., 2014). Additionally, positive results were found in healthy elderly adults, whereas a significant improvement was found in accuracy of a verbal WM task due to anodal tDCS as compared to sham tDCS (Park et al., 2014). Unfortunately, the selected studies of this review did not find any of these findings in the ABI population except for 1 study (Jo et al., 2009). On the other hand, it is important to note that a healthy or an aging brain could possibly react very differently compared to the restoration mechanism of a damaged brain post ABI.

To summarize, on the basis of this review CBCR is considered the most promising novel approach of the last decade in view of the positive results and the high methodological quality of the studies. The number of studies representing VR was limited and the methodological quality low, therefore the results should be considered preliminary. The studies representing NBS did not find evidence that the use of NBS could improve memory function and these studies were considered of moderate to high quality. Therefore, on the basis of the knowledge available we recommend CBCR as promising *remediation*-oriented intervention to improve memory function post ABI.

This review stresses some important limitations of the literature available on *remediation*-oriented memory interventions after ABI. First, the ability to benefit of those techniques may vary depending on what kind of injury the individual suffers from Fish et al. (2008). This review focused on memory impairment in a heterogeneous ABI patient population group with different injury-related diagnoses. This could be considered as a limitation, as each brain injury has a different pathology (e.g., focal vs. diffuse) and different demographics (e.g., age), which result in different restoration mechanisms such as different time courses and magnitudes of recovery. TBI is associated with a hallmark pattern of pathology concerning direct damage to frontal and temporal lobes, plus diffuse axonal injury resulting from tearing and shearing mechanisms (Levine et al., 2006). This causes a reduction in gray and white matter and impairs connectivity. The focal damage resulting from stroke is more diverse. In addition to a difference in pathology, the demographics of TBI and stroke are divergent. Stroke primarily affects people over 65 years of age, whereas TBI incidence is highest in the 15–24 age groups. This difference in pathology and demographics result in different restoration mechanism. It would therefore have been preferable to make a distinction between TBI and stroke. However, we believe that selecting the ABI population for this review gave the possibility to collect a wider range of knowledge.

Second, sample size is a crucial issue in quantitative research which seeks to make statistically based generalizations from the study results to the wider ABI population. The sample sizes used in the available literature may be considered too small to draw firm conclusions. As well as the regular absence of a control condition, the lack of blinding of participants and researchers, and the explorative character of several studies. These limitations restricted the reliability of the study's conclusions and consequently restricted the ability for us to draw well-founded conclusions.

A major strength of our review is the inclusion of 15 studies that had not been evaluated by previous reviews. On the other hand a limitation of this review may be the selection of appropriate search terms. We only searched on the terms *Acquired Brain Injury, (Traumatic) Brain Injury*, or *Stroke*. This selection of search terms may be quite limited, as ABI is a collective term for many more injury-related diagnoses. The collective term ABI can be subdivided into two categories: traumatic brain injury (TBI; i.e., external force traumatically injures the brain due to accidents, assaults or neurosurgery) or non-traumatic injury derived from either an internal source (NTBI; stroke, brain tumor) or an external source (e.g., poisoning, substance abuse). The selected search terms may have failed to cover the complete ABI population, even if the majority of ABI is caused by TBI or stroke. It might therefore be possible that we missed relevant studies.

A second limitation of this review may be the selection of the inclusion criteria. Solely studies evaluating interventions, with the focus primarily on improving the memory function were selected. Consequently, several studies using neuropsychological memory assessments as included outcome measure were excluded, since the primary outcome measure was variant (e.g., depression). These studies were excluded as they did not met our inclusion criteria, although their findings might have been possibly relevant to our review.

A final limitation may be the fact that we did not focus on pharmacological interventions, even though medication could be considered a *remediation*-oriented intervention. The included pharmacological interventions could have had favorable and interesting effects on the memory function. Hence, this should be considered in future research. However, in case of this particular review, we feel that pharmacological therapies were not suitable for targeting only the memory function without affecting other cognitive function.

In conclusion, the research on *remediation*-oriented interventions reviewed in this study represents just the beginning of a new research field that explores innovative possibilities for enhancing memory function in ABI patients. Even though CBCR in particular shows great promises, more research is needed to establish this *remediation*-oriented program as standard intervention in clinical practice, especially given the heterogeneity of ABI, time course of spontaneous recovery, timing of training after ABI, and generalization of effects at several levels of functioning. Although replication studies may seem less appealing, they are sorely needed in this field where many topics are novel and risk to remain novel (Fasotti and van Kessel, 2013).

## Author contributions

Conception and design of the work: Lauriane A. Spreij, Johanna M. A. Visser-Meily, Caroline M. van Heugten, Tanja C. W. Nijboer. Acquisition of data: Lauriane A. Spreij. Analysis of data: Lauriane A. Spreij, Tanja C. W. Nijboer. Interpretation of data: Lauriane A. Spreij, Tanja C. W. Nijboer. Drafting and revising the work: Lauriane A. Spreij, Johanna M. A. Visser-Meily, Caroline M. van Heugten, Tanja C. W. Nijboer. Final approval of the version to be published: Lauriane A. Spreij, Johanna M. A. Visser-Meily, Caroline M. van Heugten, Tanja C. W. Nijboer. Agreement to be accountable for all aspects of the work: Lauriane A. Spreij, Johanna M. A. Visser-Meily, Caroline M. van Heugten, Tanja C. W. Nijboer.

### Conflict of interest statement

The authors alone are responsible for the content and writing of this review. The authors declare that the research was conducted in the absence of any commercial or financial relationships that could be construed as a potential conflict of interest.

## Abbreviations

ABI: Acquired Brain Injury
TBI: Traumatic Brain Injury
WM: Working Memory
VR: Virtual Reality
CBCR: Computer-Based Cognitive Retraining
NBS: Non-Invasive Brain Stimulation
TMS: Transcranial Magnetic Stimulation
rTMS: repetitive Transcranial Magnetic Stimulation
tDCS: transcranial Direct Current Stimulation.
